# Effects of lactic acid bacteria fermented feed and three types of lactic acid bacteria (*L. plantarum*, *L. acidophilus*, *B. animalis*) on intestinal microbiota and T cell polarization (Th1, Th2, Th17, Treg) in the intestinal lymph nodes and spleens of rats

**DOI:** 10.5713/ab.22.0301

**Published:** 2022-11-14

**Authors:** Da Yoon Yu, Sang-Hyon Oh, In Sung Kim, Gwang Il Kim, Jeong A Kim, Yang Soo Moon, Jae Cheol Jang, Sang Suk Lee, Jong Hyun Jung, Hwa Chun Park, Kwang Keun Cho

**Affiliations:** 1 Division of Animal Science, Gyeongsang National University, Jinju 52725, Korea; 2 Division of Animal Bioscience & Integrated Biotechnology, Gyeongsang National University, Jinju 52725, Korea; 3 Department of Animal Science and Technology, Sunchon National University, Sunchon 57922, Korea; 4 Jung P&C Institute, Yongin 16950, Korea; 5 Dasan Genetics, Namwon 55716, Korea

**Keywords:** Body Weight, Intestinal Microbiota, Lactic Acid Bacteria, *Rubus coreanus*, T Cell Polarization

## Abstract

**Objective:**

In this study, we investigated the effects of *Rubus coreanus*-derived lactic acid bacteria (LAB) fermented feed (*RC*-LAB fermented feed) and three types of LAB (*Lactobacillus plantarum*, *Lactobacillus acidophilus*, *Bifidobacterium animalis*) on the expression of transcription factors and cytokines in Th1, Th2, Th17, and Treg cells in the intestinal lymph nodes and spleens of rats. In addition, the effect on intestinal microbiota composition and body weight was investigated.

**Methods:**

Five-week-old male rats were assigned to five treatments and eight replicates. The expression of transcription factors and cytokines of Th1, Th2, Th17, and Treg cells in the intestinal lymph nodes and spleens was analyzed using real-time reverse transcriptase polymerase chain reaction assays. Intestinal tract microbiota compositions were analyzed by next-generation sequencing and quantitative polymerase chain reaction assays.

**Results:**

*RC*-LAB fermented feed and three types of LAB increased the expression of transcription factors and cytokines in Th1, Treg cells and Galectin-9, but decreased in Th2 and Th17 cells. In addition, the intestinal microbiota composition changed, the body weight and Firmicutes to Bacteroidetes (F/B) ratio decreased, and the relative abundance of LAB increased.

**Conclusion:**

LAB fermented feed and three types of LAB showed an immune modulation effect by inducing T cell polarization and increased LAB in the intestinal microbiota.

## INTRODUCTION

The gut microbiota phylogenic diversity of animals has 375 phylotypes for pigs, 300 to 1,000 bacteriological specifications for cow rumen, approximately 915 operational taxonomic units for chickens, and 2,000 to 3,000 operational taxonomic units for sheep [1]. The dominant bacteria phyla of mammals are Firmicutes and Bacteroidetes, which account for 90% of the total bacteria. There are more than 274 genera in the phylum Firmicutes, including *Bacillus*, *Lactobacillus*, *Mycoplasma*, and *Clostridium*. There are about 20 genera of phylum Bacteroidetes, of which the absolute genus in the gastrointestinal tract is *Bacteroides* [2]. Gut microbiota plays an essential role in the absorption, storage, and expenditure of the energy obtained from dietary intake.

Lactic acid bacteria (LAB) as probiotics are non-spore-forming, Gram-positive rods or cocci that are resistant to low pH. They are classified as Phylum Firmicutes, Class Bacilli, and Order Lactobacillales according to cellular morphology and glucose fermentation mode. Their genera include *Lactobacillus*, *Streptococcus*, *Leuconostoc*, *Carnobacterium*, *Lactococcus*, *Aerococcus, Enterococcus*, *Pediococcus*, *Oenococcus*, Weissella, *Alloiococcus*, *Tetragenococcus*, *Dolosigranulum*, and *Vagococcus* [3].

The most commonly used bacteria genera in probiotics formulations include *Lactobacillus*, *Enterococcus*, *Streptococcus*, *Bacillus*, and *Bifidobacterium*, as well as some fungal strains of *Saccharomyces* genus such as *Saccharomyces boulardii* (*S. boulardii*) [4]. Probiotics regulate innate and adaptive immunity, promoting the immune system’s development and maturation, and enhancing the viability of macrophages and natural killer cells [5]. Dietary fiber and short-chain fatty acids (SCFAs) support the activity of T helper cells, T cytotoxic cells, Tregs, and B cells. The main function of SCFA is to increase protein acetylation and cellular metabolism in T and B cells. This affects the naive T-cell differentiation to Th1 cells, Th17 cells, and Tregs [6]. Th1-mediated responses by probiotics upregulate downstream signaling molecules T-box expressed in T cells (T-bet), signal transducer and activator of transcription-1 (STAT-1), and STAT-4, as well as interleukin-2 (IL-2) and interferon-gamma (IFN-γ). Conversely, the downregulations of GATA binding protein 3 (GATA-3), C-maf, IL-4, IL-5, and IL-17 inhibit the Th2 and Th17 responses and regulate the immune response [7].

*Rubus coreanus Miquel* (*R. coreanus*) fruit contains flavonoids, anthocyanins, tannins, quercetins, minerals, and vitamins. It is also rich in phenolic compounds such as ellagic acid (EA), gallic acid, cinnamic acid, protocatechuic acid, sanguiin H-4, sanguiin H-6, 23-hydroxytormentic acid, and niga-ichgoside F [8]. Our group has investigated the effect of *R. coreanus* byproducts on intestinal microorganisms and the immune system to confirm their efficacy as animal feed additives [9]. In this study, the effects of *R. coreanus* byproducts fermented with *R. coreanus*-derived LAB (*L. plantarum* GBL 16 and 17) and three types of LAB (*L. plantarum*, *L. acidophilus*, *B. animalis*) on the body weight, immunomodulation, and intestinal microbiome composition of rats were investigated.

## MATERIALS AND METHODS

All experimental protocols involving animals in the present study were approved by the Institutional Animal Care and Use Committee (IACUC) of Gyeongnam National University of Science and Technology (No. 2017-7).

### Animals and diets

*R. coreanus* byproducts were prepared according to the method of Yu et al [9], and *L. plantarum* GBL16 (Accession No. KCCM11621p) and 17 (Accession No. KCCM11622p) separated from *R. coreanus* were cultivated two or more times in de Man, Rogosa and Sharpe (MRS) medium (Difco, Detroit, MI, USA) [10]. Twenty percent of LAB (*L. plantarum* GBL 16 and 17, 10^9^ CFU/g) culture MRS broth was added to feed consisting of *R. coreanus* byproducts, molasses, wheat gluten, corn, and soybean meal, and then incubated at 25°C± 3°C for 4 to 5 days, dried to have less than 12% moisture content, ground up and used as *R. coreanus*-derived LAB fermented feed (*RC*-LAB fermented feed).

Five-week-old male rats (Sprague-Dawley) were purchased from Samtako (Osan, Korea), and eight were placed in each treatment group. Experiments were conducted with six treatment groups which were the basal diet (AIN-76, Feed Lab, Guri, Korea) feeding group (control group; C); the basal diet with 1% *RC*-LAB fermented feed feeding group (T1); the basal diet with 2% *RC*-LAB fermented feed feeding group (T2); the basal diet with 2% *L. plantarum* feeding group (T3); the basal diet with 2% *L. acidophilus* feeding group (T4); and the basal diet with 2% *Bifidobacterium animalis* (*B. animalis*) feeding group (T5).

The temperature and the relative humidity of the animal housing were maintained at 22°C±3°C and 55%±5%, respectively, and the light and dark cycles were 12 hours. The rats were fed the diets mentioned above for eight weeks on *ad libitum* feeding after the 7-day acclimatization period and always had free access to water. Weight and feed intake were recorded once a week during the experimental period.

### DNA extraction and next-generation sequencing

To analyze microbiota composition in the intestinal tract, fecal samples were collected after all rats were euthanized with diethyl ether and stored at −80°C for genomic DNA (gDNA) extraction. DNA was extracted using Fecal DNA MiniPrep Kits (Zymo Research, Irvine, CA, USA), and the sequence was analyzed using next-generation sequencing (NGS; Illumina MiSeq; Chunlab Inc., Seoul, Korea) and CLcommunity software [11]. We compared through literature review six types of pathogenic microorganisms (*Clostridium*, *Bacteroides*, *Prevotella*, *Mollicutes*, *Porphyromonas*, and *Campylobacter*), six types of obesity microorganisms, six types of antiobesity microorganisms (*Roseburia*, *Oscillibacter*, *Mollicutes*, *Coprobacillus*, *Allobaculum*, and *Faecalibaculum*), six types of LAB (*Lactobacillus*, *Subdoligranulum*, *Streptococcus*, *Bifidobacterium*, *Enterococcus*, and *Pediococcus*), and 11 types of butyric acid bacteria (*Eisenbergiella*, *Propionibacterium*, *Eubacterium*, *Pseudoflavonifractor*, *Ruminococcus*, *Roseburia*, *Butyricicoccus*, *Coprococcus*, *Subdoligranulum*, *Fusobacterium*, and *Butyrivibrio*) by classifying them into functional microorganism.

### Quantitative polymerase chain reaction and real-time reverse transcriptase polymerase chain reaction

Rotor-Gene SYBR Green PCR Kits and Rotor-Gene Q (Qiagen, Hilden, Germany) were used for quantitative polymerase chain reaction (qPCR) to analyze changes in major intestinal microflora. To investigate the effect on T-cell polarization, transcription factors, cytokine, and expression of Galectin-9 were analyzed. The methods suggested by Yu et al [9] were used for the separation and analysis of RNA from spleen and mesenteric lymph nodes (MLNs), all primers, qPCR, and real-time reverse transcriptase polymerase chain reaction (RT-PCR) cycling conditions. Glycolytic glyceraldehyde-3-phosphate dehydrogenase was used for normalization. To analyze the effect of *RC*-LAB fermented feed and three types of LAB on T cell polarization, the expression of transcription factors (T-bet for Th1, GATA 3 for Th2 cells, RAR-related orphan receptor gamma T [RORγT] for Th17 cells, and forkhead box P3 [Foxp3] for Treg cells), cytokines (IFN-γ for Th1 cells, IL-4 for Th2 cells, and IL-17 for Th17 cells and IL-10 for Treg cells), and Galectin-9 were measured.

### Statistical analysis

All results were represented by mean±standard deviation, and SPSS Statistics v20 (IBM, Armonk, New York, USA) was used to analyze Duncan's multiple range test at p<0.05 level.

## RESULTS

### Body weight, daily gain, daily feed intake, and feed efficiency in rats

Body weight, daily gain, daily feed intake and feed efficiency were analyzed to investigate the effects of *RC*-LAB fermented feed and three types of LAB (*L. plantarum*, *L. acidophilus*, *B. animalis*) on the growth of the rats (Table 1). T1 showed no difference in average daily gain (ADG), average feed intake (ADFI), and feed efficiency (FE) compared to C.

However, T1 showed higher ADG and FE compared to other LAB treatment groups (T3, T4, T5), including T2 (p< 0.05). T2 showed no difference in ADFI, but had significantly lower FE and ADG compared to T1 (p<0.05). In particular, T2, T4, and T5 had substantially lower ADG than C and T1 (p<0.05). As a result, T1 maintained a similar ADFI and FE to C, increasing ADG.

### Th1, Th2, Th17, Treg cells, and Galectin-9 activity in MLN and spleen

**Figure 1:**
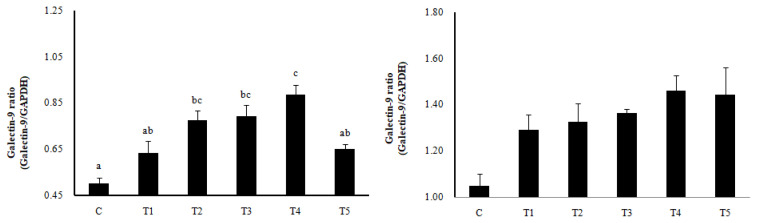
Galectin-9 expression in mesenteric lymph nodes (MLN, left) and spleen (right) by RT-PCR analysis. Data represent means±standard deviations of 8 replicates. *RC*-LAB, *Rubus coreanus*-derived lactic acid bacteria; C, control; T1, 1% *RC*-LAB fermented feed; T2, 2% *RC*-LAB fermented feed; T3, 2% *L. plantarum*; T4, 2% *L. acidophilus*; T5, 2% *B. animalis*. ^a–c^ Means are significantly different within the same row (p<0.05).

**Figure 2:**
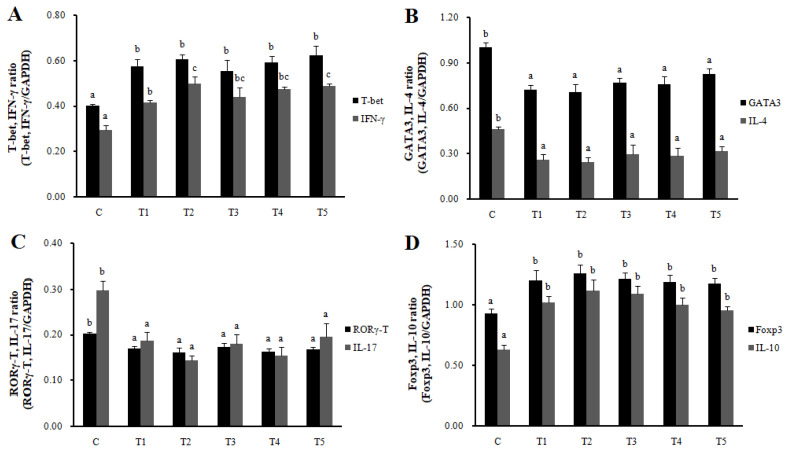
Th1, Th2, Th17, and Treg cells transcription factors and cytokines expression in MLN by real-time reverse transcriptase polymerase chain reaction analysis. T-bet, IFN-γ (Th1, A), GATA-3, IL-4 (Th2, B), RORγT, IL-17 (Th17, C), and Foxp3, IL-10 (Treg, D). Data represent means±standard deviations of 8 replicates. *RC*-LAB, *Rubus coreanus*-derived lactic acid bacteria; T-bet, T-box expressed in T cells; GATA 3, GATA binding protein 3; RORγT, RAR-related orphan receptor gamma T; Foxp3, forkhead box P3; IFN-γ, interferon-gamma; IL-4, interleukin-4; C, control; T1, 1% *RC*-LAB fermented feed; T2, 2% *RC*-LAB fermented feed; T3, 2% *L. plantarum*; T4, 2% *L. acidophilus*; T5, 2% *B. animalis*. ^a–c^ Means are significantly different within the same row (p<0.05).

**Figure 3:**
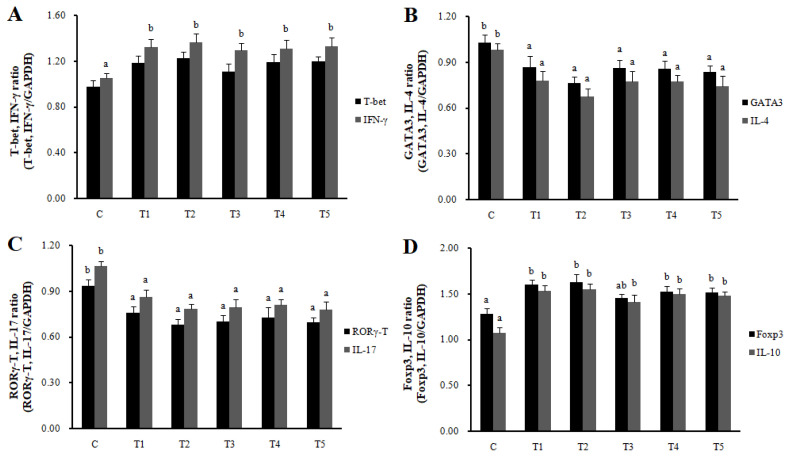
Th1, Th2, Th17, and Treg cells transcription factors and cytokines expression in spleen by real-time reverse transcriptase polymerase chain reaction analysis. Data represent means±standard deviations of 8 replicates. *RC*-LAB, *Rubus coreanus*-derived lactic acid bacteria; T-bet, T-box expressed in T cells; IFN-γ, interferon-gamma; GATA 3, GATA binding protein 3; IL-4, interleukin-4; RORγT, RAR-related orphan receptor gamma T; Foxp3, forkhead box P3; C, control; T1, 1% *RC*-LAB fermented feed; T2, 2% *RC*-LAB fermented feed; T3, 2% *L. plantarum*; T4, 2% *L. acidophilus*; T5, 2% *B. animalis*. T-bet, IFN-γ (Th1, A), GATA-3, IL-4 (Th2, B), RORγT, IL-17 (Th17, C) and Foxp3, IL-10 (Treg, D). ^a–c^ Means are significantly different within the same row (p<0.05).

RNA expression of MLN Galectin-9 gene, known to induce the immune response of Treg cells, was increased in T2, T3, and T4 (LAB groups) compared to C (p<0.05) (Figure 1). The RNA expression of the Galectin-9 gene in the spleen showed a tendency to increase in all treatment groups compared to C. In MLN, the cytokine IFN-γ and the transcription factors of Th1 cells increased in all treatment groups compared to C (p<0.05), and the cytokine IL-4 and the transcription factors GATA-3 of Th2 cells decreased in all treatment groups compared to C (p<0.05). Cytokine IL-17 and the transcription factors RORγ-T in Th17 cells were reduced in all treatment groups compared to C (p<0.05), and cytokine IL-10 and the transcription factors Foxp3 in Treg cells were increased in all processing ports compared to C (p<0.05) (Figure 2). Also, in the spleen, like with MLN, the cytokines and the transcription factors of Th1 and Treg cells increased in all treatment groups compared to C, and those of Th2 and Th17 cells decreased in all treatment groups compared to C (p<0.05) (Figure 3). Cytokines and the transcription factors of Th1 and Treg cells increased the transcription level, while those of Th2 and Th17 cells decreased the level. Therefore, it was found that the LAB treatments regulate immunity in rats. The expression of MLN Galectin-9 was significantly increased in all treatment groups compared to C (Figure 1) (p<0.05). Also, the expression of spleen Galectin-9 showed a tendency to rise in all treatment groups compared to C.

### Changes in gut microbiota composition by next-generation sequencing

**Figure 4:**
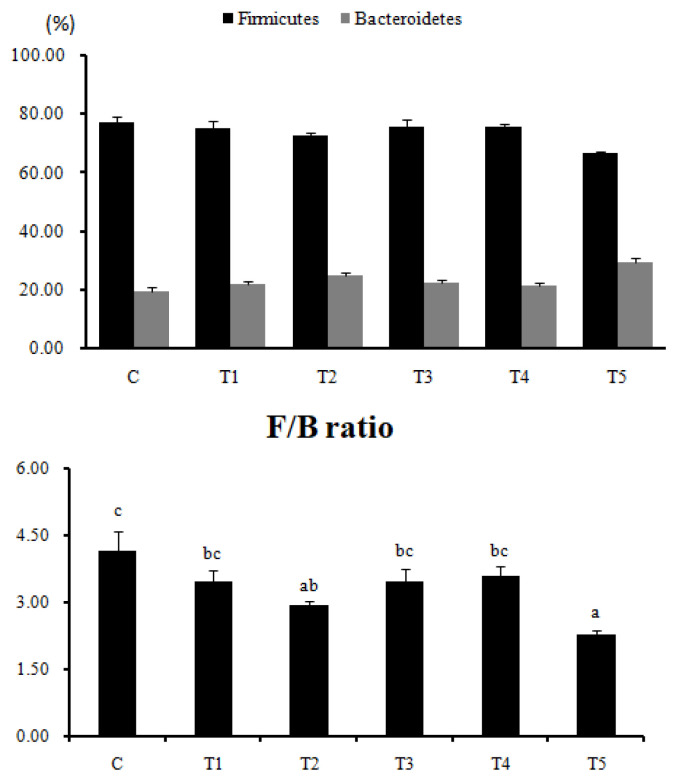
Influence of *RC*-LAB fermented feed and 3 types of LAB on the relative abundance of phyla Firmicutes and Bacteroidetes in the digestive tract of rats by next generation sequencing (Illumina MiSeq; Chunlab Inc., Seoul, Korea). *RC*-LAB, *Rubus coreanus*-derived lactic acid bacteria. Data represent means±standard deviations of 8 replicates. C, control; T1, 1% *RC*-LAB fermented feed; T2, 2% *RC*-LAB fermented feed; T3, 2% *L. plantarum*; T4, 2% *L. acidophilus*; T5, 2% *B. animalis*. ^a–c^ Means are significantly different within the same row (p<0.05).

The effect of *RC*-LAB fermented feed and three types of LAB feeding on the gut microbiota composition in rats was investigated (Table 2, Figure 4). At the phylum level, Firmicutes and Bacteroidetes account for more than 90% of the gut microbiota composition in the control and the LAB treatment groups, indicating that they are the dominant bacteria among the intestinal microorganisms. The F/B ratio was the lowest in T2 and T5, which showed lower ADG compared to the control (p<0.05). The phylum Firmicutes showed a tendency to decrease in all treatment groups compared to C, but the phylum Bacteroidetes tended to increase in all treatment groups compared to C (Table 2). Class Clostridia and family Romboutsia of phylum Firmicutes were significantly lower in T2 and T5, showing the lowest ADG compared to the basic diet of the control group (p<0.05). On the other hand, family Eisenbergiella of class Clostridia increased in all treatment groups compared to C, and family Eubacterium_g17 increased in T1, T2, T3, and T4 (p< 0.05). Class Bacilli of phylum Firmicutes decreased in all treatment groups compared to C (p<0.05), and family Lactobacillus of class Bacilli was the lowest in T2 and T5 showing a low ADG (p<0.05). Class Bacteroidia of phylum Bacteroides increased in all treatment groups compared to C (p< 0.05), and family Prevotella of class Bacteroidia increased in T1, T2, T3, and T4 compared to C (p<0.05). Lastly, Alistipes and S24-7_f_uc showed an increasing tendency in all treatment groups compared to C.

### Changes in pathogen, obesity, antiobesity, lactic acid, and butyric acid bacteria in the gut microbiota composition by next-generation sequencing

**Figure 5:**
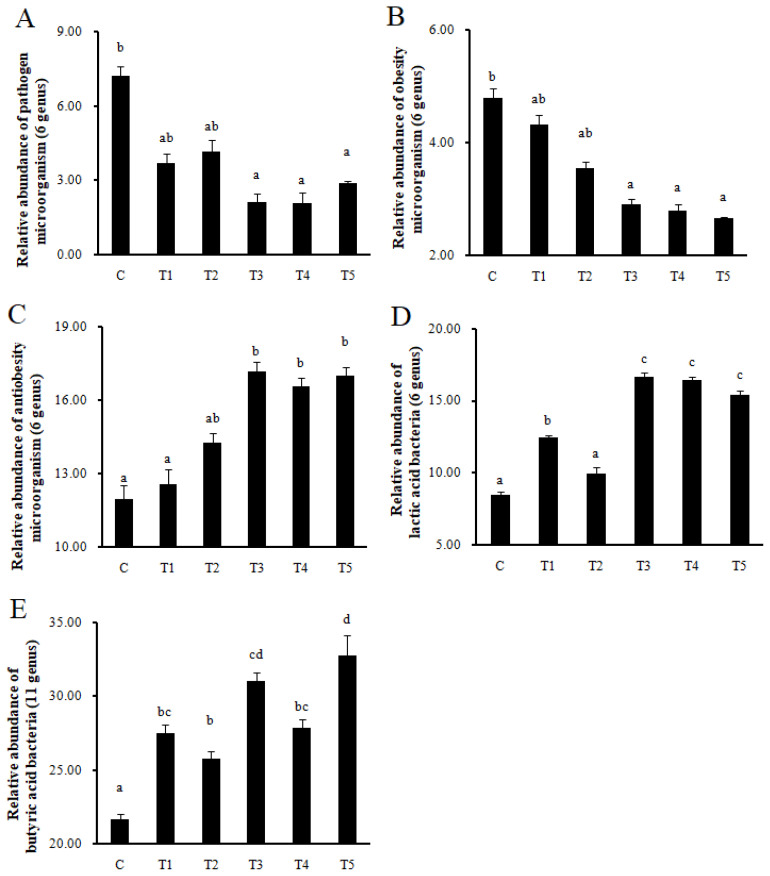
Comparison of the relative abundance in 6 types of pathogen bacteria, 6 types of obesity bacteria, 6 types of antiobesity bacteria, 6 types of lactic acid bacteria and 11 types of butyric acid bacteria in the gut microbiota composition by next generation sequencing (Illumina MiSeq; Chunlab Inc., Seoul, Korea). C, control; T1, 1% *RC*-LAB fermented feed; T2, 2% *RC*-LAB fermented feed; T3, 2% *L. plantarum*; T4, 2% *L. acidophilus*; T5, 2% *B. animalis*. (A) 6 types of pathogen bacteria (*Clostridium*, *Bacteroides*, *Prevotella*, *Mollicutes*, *Porphyromonas*, *Campylobacter*); and (B) 6 types of obesity bacteria (*Roseburia*, *Oscillibacter*, *Mollicutes*, *Coprobacillus*, *Allobaculum*, *Faecalibaculum*); and (C) 6 types of antiobesity bacteria (*Prevotella*, *Alistipes*, *Bacteroides*, *Prevotellaceae*, *Bacteroides*, *Parabacteroides*); and (D) 6 types of lactic acid bacteria (*Lactobacillus*, *Subdoligranulum*, *Streptococcus*, *Bifidobacterium*, *Enterococcus*, *Pediococcus*); and (E) 11 types of butyric acid bacteria (*Eisenbergiella*, *Propionibacterium*, *Eubacterium*, *Pseudoflavonifractor*, *Ruminococcus*, *Roseburia*, *Butyricicoccus*, *Coprococcus*, *Subdoligranulum*, *Fusobacterium*, *Butyrivibrio*). *RC*-LAB, *Rubus coreanus*-derived lactic acid bacteria. Data represent means±standard deviations of 8 replicates. ^a–d^ Means are significantly different within the same row (p<0.05).

Pathogens and obesity microorganisms decreased in T3, T4, and T5 compared to C (p<0.05), while antiobesity microorganisms increased in T3, T4, and T5 (p<0.05). In addition, LAB increased in T1, T3, T4, and T5 compared to C (p<0.05), and butyric acid bacteria also increased in all treatment groups compared to C (p<0.05) (Figure 5).

### Changes in major intestinal microflora by qPCR analysis

The qPCR was performed using 12 types of major intestinal microflora after the completion of feeding trials to analyze the effect of *RC*-LAB fermented feed and three types of LAB feeding on the major intestinal microflora (Table 3). In the treatment groups, potential colonizers *Bacteroides* spp., which make up a significant fraction of the gut bacteriome [12], showed an increasing tendency compared to C in the qPCR as well as in the results of the NGS as shown in Table 2. In particular, there was a significant increase in T5, which lost the most weight (p<0.05).

There was no difference between the control group and treatment groups for *Roseburia* spp., *Faecalibacterium prausnitzii*, *Ruminococcus* spp. in SCFAs-producing bacteria, and *Weissella koreensis* in LAB. *Bifidobacterium* spp., *Methanogens*, *Oscillospira* spp., *Leuconostoc citreum*, and *Weissella cibaria* increased significantly in the treatment groups (p<0.05), and *Bifidobacterium* spp. increased in T5 the most (p<0.05). In addition, *Leuconostoc mesenteroides* significantly increased in T3, T4, and T5 compared to C (p<0.05). *Lactobacillus sakei* increased in the *Lactobacillus* groups (T1, T2, T3, and T4) except for the *Bifidobacterium lactis* group (T5) compared to C (p<0.05), especially in T3 and T4 (p<0.05).

## DISCUSSION

Among the major active compounds of *R. coreanus*, the EA having antiobesity and antioxidant properties is metabolized and absorbed into urolithins (3,4-benzocoumarin derivatives) by microbial fermentation. In high-fat diet (HFD)-induced obese C57BL/6 mice, *R. coreanus*-derived EA is effective in inhibiting body weight gain and improving lipid profile [13]. In the results of this study, there was no difference in ADFI between T1 and T2, but the reason that FE and ADG were lower in T2 is presumed to be the antiobesity effect of EA (Table 1).

Hric et al [14] investigated the effect of consuming LAB-rich (such as *Lactococcus*, *Streptococcus*, *Lactobacillus*, and *Enterococcus*) Bryndza cheese on middle-aged women's intestinal microorganisms and on short weight loss programs (4 weeks). It was reported that the relative abundance of LAB (*Lactobacillales*, *Streptococcaceae*, *Lactococcus*, and *Streptococcus*) significantly increased in the intestines of women who ingested Bryndza cheese, and short-chain fat acid producers also significantly increased, improving body composition (body weight, height, body fat percentage, amount of fat mass and muscle mass).

Takahashi et al [15] evaluated the effect of *Bifidobacterium animalis* ssp. *lactis* GCL2505 (*B. lactis* GCL2505) on abdominal visceral fat storage for overweight and mildly obese Japanese adults. Subcutaneous abdominal fat areas significantly decreased in the *Bifidobacterium animalis* ssp. *lactis* group, but the total number of fecal *bifidobacteria* increased dramatically. They said that *Bifidobacterium animalis* ssp. *lactis* could act as a specific functional food to achieve the visceral fat reduction of overweight or mildly obese indications. *Bifidobacterium animalis* ssp. increases the gut SCFA levels of mice and shows anti-metabolic syndrome effects through the SCFA receptor G protein-coupled receptor 43 (GPR43).

Sousa et al [16] reported that the direct administration of *Lactobacillus* supernatant into the rat central nervous system resulted in a decrease in body weight and an increase of the leptin expression in a specific area of the brain and retroperitoneal adipose tissue.

Cheng and Liu [17] stated that feeding a HFD to mice increased the proportion of Proteobacteria and the ratio of Firmicutes/Bacteroidetes of the microbiota. However, HFD with a high-dose *Lactobacillus rhamnosus* GG (LGG) restored exogenous leptin responsiveness, increased the ratio of villus height to crypt depth, and reduced the proportion of the Proteobacteria in the fecal microbiota.

Supplementing *Lactobacillus rhamnosus* fermented milk to old mice has been shown to alleviate the immunosenescence-associated Th1/Th2 imbalance and improve antioxidant capacity [18]. Oral administration of *L. paracasei* KBL 382 decreased the production of Th1, Th2, and Th17 type cytokines in skin issues and increased the production of the anti-inflammatory cytokine IL-10 [19]. It also increased the proportion of CD4^+^ CD25^+^ Foxp3^+^ regulatory T cells in MLNs. Furthermore, it dramatically changed the gut microbiota composition of atopic dermatitis (AD) mice and alleviated AD-like symptoms by controlling the immune response.

*Bifidobacteria* is one of the probiotics used to treat intestinal diseases. *B. infantis* increases the level of Th1 cytokines in mice and decreases the level of Th2 cytokines resulting in having the therapy effects of allergic asthma. In addition, *Bifidobacterium pseudocatenulatum* CECT 7765 restores the balance between regulatory T cells (Tregs) and B lymphocytes, and reduces the adaptive (IL-17A) and innate tumor necrosis factor-α (TNF-α) immunity and the pro-inflammatory cytokines of endotoxemia resulting in the reduction of obesity-associated systemic inflammation [20]. Bifidobacterial administration restores HFD-induced alternations in the microbiota. In other words, it reduces body weight gain and the abundance of Firmicutes and LPS-producing Proteobacteria. Immune cellular mechanisms identified that the administration of a specific *Bifidobacterium* strain weakened the inflammatory cascade associated with diet-induced obesity, which is relevant to regulating the gut microbiota structure. *Bifidobacteria* settle in the human gastrointestinal tract immediately after birth when interaction with the host begins [21]. The health benefits of *Bifidobacteria* vary from strain to strain and may be due to polysaccharide production. Probiotics intake can prevent obesity, irritable bowel syndrome, eczema or atopic dermatitis, and asthma. *Bifidobacteria* and *lactobacilli* can be used as a prophylactic treatment for enteric pathogens, antibiotic-associated diarrhea, lactose intolerance, ulcerative colitis, irritable bowel syndrome, colorectal cancer, cholesterol reduction, and for controlling obesity and metabolic disorders.

Galectins are the soluble form of lectins and show specific binding activity for beta-galactoside sugars. Galectin-1, -2, -3, -4, -9 are typically expressed in the gastrointestinal tract, and are involved in the regulation of intestinal homeostasis and immunity. In particular, galectin-9 directly binds to CD44 (single-pass transmembrane glycoprotein) to promote Foxp3 expression of Tregs, which inhibits exclusive Th2 responses [22].

YK4 (probiotic mixture consisting of *Lactobacillus acidophilus* CBT LA1, *L. plantarum* CBT LP3, *Bifidobacterium breve* CBT BR3, and *B. lactis* CBT BL3) inhibits the expression of skin thymic stromal lymphopoietin and serum immunoglobulin E induced by the excessive Th 2 cell-mediated responses of mice, thus mitigating AD [23]. YK4 also inhibits the Th2 cell population by inducing proportions of Th1 cells in the spleen and proportions of Treg cells in Peyer’s patches and MLNs. CD103+ dendritic cells (DCs) treated with YK4 induce differentiation of naïve T cells toward Th1 and Tregs to inhibit the Th2 response and increased galectin-9 expression in the gut.

A specific oligosaccharide mixture can control the immune response by improving the galectin-9 levels. Galectin-9 induces the polarization of Th1 and Treg cells in the presence of bacterial-derived CpG DNA or synthetic toll-like receptor 9 (TLR9) ligand CpG-oligonucleotide (ODN). Immunostimulatory properties are associated with high GC content (unmethylated CpG motifs) preserved in the bacterial genome, where CpG motif activates TLR9 and induces Th1-type immune responses. CpG motifs are more present in prokaryotes (95% of CpG motifs are unmethylated) than in eukaryotes (10 to 30% of CpG motifs are unmethylated). *Bifidobacteria* has an average GC content of 60.1% whereas the average GC content of *Lactobacillus* is 46.61%, so *Bifidobacteria* has a 14.49% higher GC content than *Lactobacillus* [24].

Species in the phyla Bacteroidetes and Firmicutes account for more than 90% of the gastrointestinal microbiota. Changes in the proportion of these two bacterial groups were found to have contrasting health effects, including obesity and inflammatory diseases. Obese mice have an increased relative abundance of phylum Firmicutes and a decreasing relative abundance of phylum Bacteroidetes, which is reversed when diet-induced weight loss occurs. As the *Firmicutes* to *Bacteroidetes* (F/B) ratio increases, the ability to ferment dietary polysaccharides into SCFA increases. According to the literature review, it is unclear whether the F/B ratio and SCFAs can be considered biomarkers for obesity. The F/B ratio and SCFAs are interrelated, as most butyrate producers are included in the phylum Firmicutes [25]. However, many more species cannot produce SCFA in Firmicutes.

*Clostridium*, a genus of phyla Firmicutes, is one of the largest prokaryotic genera, including the heterogeneous group of rod-shaped, anaerobic and spore-forming bacteria. The importance of this genus is reflected by more than 42,000 entries in the PubMed database and by approximately 1,700 genome sequences regarding this group deposited in the GenBank database. This group includes important human and animal pathogens such as *C. botulinum*, *C. tetani*, and *C. difficile*, and industrially relevant microorganisms such as *C. acetobutylicum*. Commensal *Clostridia* has the bile acid-inducible (bai) operon, which allows for the conversion of cholate (CA) to deoxycholate (DCA) and the gut microbiota-derived secondary bile acids DCA provides colonization resistance to *Clostridioides difficile* [26].

The genus *Eubacterium* is a core human gut microbiome and plays an important role in supporting energy homeostasis, colonial motility, immunomodulation, and the suppression of gut inflammation. Many species of the genus *Eubacterium* are considered promising targets of microbial therapeutics. Specific strains, especially butyrate-producing microbes that belong to genera *Eubacterium*, *Roseburia*, and *Faecalibacterium*, ultimately benefit animal health in the same way as strains in Lactobacillus and *Bifidobacterium* [27].

Genus *Lactobacillus* are rod-shaped, gram-positive, non-spore-forming, facultative anaerobic bacteria in the phylum Firmicutes. *Lactobacillus* is the largest genus in the LAB group that makes carbohydrates into lactic acid [28].

Phylum *Bacteroidetes* are rod-shaped gram-negative bacteria rich in animal guts and skins as well as the environment, and can make up more than 60% of microbial communities. Members of the phylum Bacteroidetes can convert intractable carbon sources into biomass and short chain fatty acids [29].

The genera *Prevotella* is one of the most dominant genera in the large intestine of pigs. Prevotella-driven enterotype shows positive associations with economically important traits in food animal production, including feed intake, feed efficiency, weight gain, and incidence of diarrhea, and plays an important role in helping *Prevotella* mediate growth performance and disease resilience [30]. *Alistipes* are anaerobic, gram-negative bacteria found in the healthy human gastrointestinal (GI) tract microbiota. *Alistipes finegoldii* can assemble membrane lipids using medium- and long-chain fatty acid nutrients available in the gut environment [31].

Genera S24-7_F_uc, *Lactobacillus*, *Ruminococcus*, *Olsenella*, *Enterohabdus*, *Sphingobacterium*, *Pseudomonas*, *Alistipes*, *Ochrobactrum*, *Bradyr-hizobium*, *Mucispiruillum*, *Acetatifactor*, and *Bacillus* show positive relationships with the markers for brain and gastrointestinal health, whereas *Clostridium*, *Adlercreutzia*, *Turicibacter*, *Enterococcus*, *Caproiciproducens*, and *Desulfovibrio* show high negative relationships with chronic stress markers such as serum corticosterone concentration, anxiety-like behaviors, proapoptotic molecules, and inflammatory mediators [32]. These results indicate that the intestinal microbiota composition is significantly affected by external chromatic stress and is related to brain function and behavioral properties. Mechanisms in which probiotics protect hosts from infections include epithelial barrier enhancement, pathogen adhesion suppression, antibacterial substance synthesis, toxins or toxin receptor modification, and non-specific and specific immune response stimuli for pathogens [33].

Changes in diversity and richness of microorganisms are associated with SCFA composition, energy homeostasis, and inflammation. However, the relationship between gut microbiota and energy homeostasis is complicated because variables such as genes, age, and diet affect gut microbiota [34].

## CONCLUSION

Our results demonstrate a role for LABs in regulating the structure and function of the intestinal microbiota. In addition, our data reveal new insights into the role of supplementing feed additives that could lead to therapeutic developments for intestinal health.

## Figures and Tables

**Table 1:** Body weight, daily gain, daily feed intake, and feed efficiency in rats by the treatment group

Items	C1)	T1	T2	T3	T4	T5
Initial body weight (g)	189.75±2.01^a^	189.65±2.01^a^	189.92±3.41^a^	187.27±3.26^a^	189.52±3.97^a^	189.82±2.60^a^
Finished body weight (g)	425.75±0.77^d^	429.40±0.60^e^	409.88±0.44^ab^	414.68±0.29^c^	410.83±0.44^b^	408.33±0.71^a^
Average daily gain (g)	4.92±0.03^bc^	4.99±0.05^c^	4.58±0.07^a^	4.74±0.07^ab^	4.61±0.09^a^	4.55±0.07^a^
Average daily feed intake (g)	22.20±0.41^bc^	21.28±0.29^ab^	21.17±0.29^a^	21.65±0.38^ab^	22.64±0.14^c^	21.84±0.28^abc^
Feed efficiency	0.22±0.004^cd^	0.23±0.004^d^	0.22±0.006^abc^	0.22±0.006^bc^	0.20±0.003^a^	0.21±0.005^ab^

*RC*-LAB, *Rubus coreanus*-derived lactic acid bacteria; SD, standard deviation; FBD, finished body weight; ADG, average daily gain; ADFI, average daily feed intake.

Data represent means±standard deviation of 8 replicates.

1) C, control; T1, 1% *RC*-LAB fermented feed; T2, 2% *RC*-LAB fermented feed; T3, 2% *L. plantarum*; T4, 2% *L. acidophilus*; T5, 2% *B. animalis*.

FBD and ADG increased when *RC*-LAB fermented feed (T1) was fed as compared to LAB treatment group. FBD and ADG were the lowest in 2% *B. animalis* (T5) among the three LAB treatment groups.

a–e Means are significantly different within the same row (p<0.05).

**Table 2:** Influence of *RC*-LAB fermented feed and 3 types of LAB on the relative abundance of dominant of phyla, classes and familia in the digestive tract of rats by next-generations sequencing

Items2)	Treatments1)					

	C	T1	T2	T3	T4	T5

	n = 8 mean%	n = 8 mean%	n = 8 mean%	n = 8 mean%	n = 8 mean%	n = 8 mean%
Firmicutes	77.04±2.31^a^	75.13±2.36^a^	72.70±1.13^a^	75.89±2.15^a^	75.77±1.13^a^	66.56±0.75^a^
Clostridia	66.16±2.51^c^	61.04±1.56^bc^	57.68±1.58^ab^	60.58±1.26^bc^	60.67±2.28^bc^	52.47±2.39^a^
Eisenbergiella	6.15±0.60^a^	10.79±0.76^bc^	9.20±0.61^b^	11.62±0.73^c^	11.17±0.76^bc^	9.38±0.68^b^
Romboutsia	3.85±0.66^c^	2.98±0.43^bc^	1.21±0.20^a^	3.37±0.51^bc^	3.60±0.38^c^	2.12±0.24^ab^
Eubacterium_g17	2.01±0.25^a^	3.85±0.42^b^	3.81±0.35^b^	4.06±0.69^b^	4.85±0.66^b^	3.31±0.37^ab^
Bacilli	17.50±1.40^c^	11.48±1.28^b^	8.42±0.83^ab^	11.62±1.19^b^	11.75±0.93^b^	6.57±0.86^a^
Lactobacillus	10.71±0.58^c^	11.19±0.64^c^	8.22±0.74^b^	11.46±0.70^c^	11.57±0.69^c^	6.25±0.47^a^
Bacteroidetes	19.27±1.43^a^	22.04±0.99^a^	24.94±0.80^a^	22.24±1.14^a^	21.46±1.13^a^	29.47±1.43^a^
Bacteroidia	13.45±1.61^a^	20.54±0.52^b^	23.77±0.83^b^	21.91±1.60^b^	19.79±1.90^b^	28.84±1.16^c^
Prevotella	5.55±0.67^a^	9.46±0.73^bc^	11.42±0.77^c^	8.61±0.79^b^	8.20±0.75^b^	7.47±0.84^ab^
Alistipes	1.89±0.40^a^	2.60±0.25^a^	2.82±0.46^a^	3.46±0.44^a^	3.32±0.35^a^	3.15±0.38^a^
S24-7_f_uc	1.63±0.25^a^	1.58±0.30^a^	2.05±0.19^a^	2.11±0.18^a^	2.19±0.32^a^	1.97±0.27^a^

Data represent means±standard deviations of 8 replicates.

*RC*-LAB, *Rubus coreanus*-derived lactic acid bacteria.

1) C, control; T1, 1% *RC*-LAB fermented feed; T2, 2% *RC*-LAB fermented feed; T3, 2% *L. plantarum*; T4, 2% *L. acidophilus*; T5, 2% *B. animalis*.

2) Statistical tests of over-or under-representation of bacterial lineages among at each sample were made at the phylum, class and family levels using Duncan's multiple range test.

a–c Means are significantly different within the same row (p<0.05).

**Table 3:** Comparison of short-chain fatty acids-producing bacteria and LAB containing gut-dominant microorganisms by quantitative polymerase chain reaction

Species	Treatments1)					

	C	T1	T2	T3	T4	T5
*Bacteroides* spp.	4.99±0.22^a^	6.42±0.56^a^	5.31±0.20^a^	6.33±0.70^a^	6.26±0.13^a^	8.72±0.64^b^
*Roseburia* spp.	5.10±0.51^a^	6.59±0.62^a^	6.05±0.41^a^	6.98±0.36^a^	6.48±0.35^a^	6.73±0.25^a^
*Faecalibacterium prausnitzii*	2.88±0.32^a^	2.95±0.12^a^	3.00±0.34^a^	3.62±0.19^a^	3.20±0.30^a^	3.23±0.15^a^
*Ruminococcus* spp.	3.16±0.19^a^	3.38±0.06^a^	3.55±0.21^a^	3.64±0.16^a^	3.75±0.23^a^	3.97±0.19^a^
*Bifidobacterium* spp.	2.54±0.14^a^	6.72±0.10^c^	5.17±0.15^b^	6.02±0.40^c^	6.38±0.27^c^	8.73±0.18^d^
*Methanogens*	1.44±0.05^a^	2.41±0.10^bc^	1.99±0.06^b^	2.47±0.10^c^	2.21±0.23^bc^	2.35±0.16^bc^
*Oscillospira* spp.	1.49±0.17^a^	2.09±0.06^b^	2.07±0.11^b^	2.49±0.12^b^	2.48±0.14^b^	2.40±0.17^b^
*Leuconostoc mesenteroides*	2.51±0.29^a^	3.01±0.12^ab^	2.92±0.20^ab^	3.19±0.14^b^	3.36±0.13^b^	3.47±0.24^b^
*Leuconostoc citreum*	2.63±0.13^a^	3.20±0.14^b^	3.09±0.13^b^	3.15±0.10^b^	3.34±0.15^b^	3.11±0.09^b^
*Weissella cibaria*	2.22±0.25^a^	6.57±0.14^b^	6.58±0.15^b^	6.28±0.16^b^	6.55±0.26^b^	6.52±0.12^b^
*Weissella koreensis*	1.76±0.18^a^	1.93±0.05^a^	1.96±0.09^a^	1.96±0.03^a^	2.10±0.07^a^	1.99±0.08^a^
*Lactobacillus sakei*	3.33±0.14^a^	4.52±0.20^bc^	4.23±0.06^b^	5.16±0.17^c^	5.12±0.20^c^	4.27±0.14^ab^

Data represent means±standard deviations of 8 replicates.

LAB, lactic acid bacteria.

1) C, control; T1, 1% *RC*-LAB fermented feed; T2, 2% *RC*-LAB fermented feed; T3, 2% *L. plantarum*; T4, 2% *L. acidophilus*; T5, 2% *B. animalis*.

a–c Means are significantly different within the same row (p<0.05).
